# Crosses with spelt improve tolerance of South Asian spring wheat to spot blotch, terminal heat stress, and their combination

**DOI:** 10.1038/s41598-021-85238-x

**Published:** 2021-03-16

**Authors:** Ajeet Kumar Pandey, Vinod Kumar Mishra, Ramesh Chand, Sudhir Navathe, Neeraj Budhlakoti, Jayasudha Srinivasa, Sandeep Sharma, Arun Kumar Joshi

**Affiliations:** 1 Department of Genetics and Plant Breeding, Institute of Agricultural Sciences, BHU, Varanasi, 221005 India; 2 Department of Mycology and Plant Pathology, Institute of Agricultural Sciences, BHU, Varanasi, 221005 India; 3 Genetics and Plant Breeding Group, Agharkar Research Institute, Pune, 411004 India; 4 ICAR- Indian Agricultural Statistics Research Institute, New Delhi, 110012 India; 5 International Maize and Wheat Improvement Centre (CIMMYT), DPS Marg, New Delhi, 110012 India; 6 Borlaug Institute for South Asia (BISA), DPS Marg, New Delhi, 110012 India

**Keywords:** Genetics, Agricultural genetics, Plant breeding

## Abstract

Spot blotch and terminal heat are two of the most important stresses for wheat in South Asia. A study was initiated to explore the use of spelt (*Triticum spelta*) to improve tolerance to these stresses in spring wheat (*T. aestivum*). We assessed 185 recombinant inbred lines (RILs) from the cross *T. spelta* (H + 26) × *T. aestivum* (cv. HUW234), under the individual stresses and their combination. H + 26 showed better tolerance to the single stresses and also their combination; grain yield in RILs was reduced by 21.9%, 27.7% and 39.0% under spot blotch, terminal heat and their combined effect, respectively. However, phenological and plant architectural traits were not affected by spot blotch itself. Multivariate analysis demonstrated a strong negative correlation between spikelet sterility and grain yield under spot blotch, terminal heat and their combination. However, four recombinant lines demonstrated high performance under both stresses and also under their combined stress. The four lines were significantly superior in grain yield and showed significantly lower AUDPC than the better parent. This study demonstrates the potential of spelt wheat in enhancing tolerance to spot blotch and terminal heat stresses. It also provides comprehensive evidence about the expression of yield and phenological traits under these stresses.

## Introduction

Wheat is a staple food crop that contributes about 20% of the total calories and protein in 89 countries across the world (https://wheat.org/wheat-in-the-world) and is critical for 2.5 billion people who live on less than US$2/day, of whom most are women and children^1^. It is extremely important for the food and nutritional security of the thickly populated region of South Asia, home to more than 1.8 billion people. The climate of the Eastern Gangetic Plains (EGP) of South Asia is characterized by high temperature and high humidity^2^. These, in turn, make the wheat crop particularly vulnerable to the two stresses—biotic (spot blotch) and abiotic (terminal heat). Spot blotch caused by fungus *Bipolaris sorokiniana* can assume epidemic proportions in the EGP region, including India, Nepal and Bangladesh. It has been reported to spread into cooler traditional rice–wheat production areas as well^3,4^. At least 17.5% yield losses has been reported in wheat due to leaf blight in the Indian subcontinent^5^. It is estimated that 10 Mha of wheat are affected by spot blotch in South Asia, of which 9 Mha are within India alone^6^. Most of it falls under the rice–wheat cropping system which often provides an environment favourable for the survival and multiplication of foliar blight pathogens, particularly due to late sowing of wheat after the preceding paddy crop^6^. Of the 220 Mha put to wheat globally, 25 Mha is affected by spot blotch^7^. Hence, this disease has become a major wheat production constraint not only in the EGP but also in warmer regions worldwide^6,8^ including North and Latin America^9^, Brazil^10^, and to some extent in parts of Europe.

Terminal heat, another major stress in wheat, is estimated to affect approximately 13.5 Mha of the wheat-growing area (~ 40% of the total irrigated area) in India alone^11^. Temperatures above 35 °C before March 30, which were uncommon in the previous century, are now a frequent occurrence in India’s EGP where spot blotch is already a dominant pathogen^2^. Wheat is very sensitive to heat stress especially at reproductive and grain filling stages^12^. Rane et al.^13^ have shown that temperatures > 30 °C at pre-and-post anthesis minimizes the rate of grain filling and thus reduces wheat production. Wheat is expected to experience one of the most severe crop yield declines from global warming, particularly related to night time temperatures in low-latitude countries^14–16^. Indeed, the Inter-governmental Panel on Climate Change^17^ has predicted that climate change alone may result in a 20% reduction in South Asia’s annual wheat production by 2030, amounting to US $7.7 billion in crop losses per year. The future is bleaker: by 2050, climate change may shrink wheat production further, by 30–40%^17^.

A significant proportion of wheat in South Asia experiences combined spot blotch and terminal heat stresses^11^. This combination is recognized as a major challenge for wheat production in the EGP^2,11^. As the most detrimental impacts of both spot blotch and terminal heat stresses coincide with the stage when wheat starts the transition from vegetative growth to grain formation, yields are severely diminished. Attributing to the fact that south Asian wheat growing season is characterised by high humidity and high temperature, it is favourable for spot blotch particularly during flowering and grain filling stage^2,18^. Heat stress and spot blotch have been found to be positively associated^19^. Moderate to warm temperature range (18 to 32 °C) generally favours the growth of spot blotch pathogen *Bipolaris sorokiniana*. Winter rainfall in south Asia is known to worsen the situation. Even in the absence of rainfall, high relative humidity arising from soil residual moisture along with foggy days (which are quite common in NEPZ) cause prolonged wetness on leaf blades and sheath that can last until late January to first fortnight of February, creating ideal conditions for the establishment and multiplication of pathogen^20^. Thus, spot blotch in combination with higher temperature at reproductive phase is even more detrimental causing increased yield losses mainly due to reduction in grain number and grain weight^21^. Reports on independent segregation of spot blotch and earliness hints at possibilities of developing early maturing wheat lines coupled with appreciable resistance to spot blotch so as to obtain high yield by employing escape mechanism against exposure to terminal heat stress^2^. Common tolerance mechanisms have sparsely been reported to combat both spot blotch and terminal heat stress.

Hence, it is imperative to manage both stresses simultaneously in the wheat breeding programs of this region. Most of earlier studies have focused on separate investigations of spot blotch or terminal heat stress in wheat, often in *T. aestivum* × *T. aestivum* crosses. Although a few studies have attempted to investigate the effects of the simultaneous stresses^11,19^, they do not provide comprehensive insights under controlled individual and combined stress conditions.

Spelt wheats show promise against abiotic stresses^22^ and generally they are more robust during the early stages of crop growth. The significance of wide hybridization has been highlighted in breeding for heat stress using *T. spelta* and other hexaploid germplasm accessions^23^. This indicates that *T. spelta* may be used as a potential source to improve the performance of wheat lines against various stresses. This appears quite logical since over a long period of domestication and breeding, wheat has lost a significant proportion of its genetic diversity and is considered to have a narrow genetic base^23^. The present study was performed to (i) identify elite lines derived from *T. spelta* (H + 26) ×  *T. aestivum* (cv. HUW234) having appreciable tolerance to spot blotch, terminal heat stress and their combination and (ii) to study the response of this population under spot blotch, terminal heat and their combination.

## Results

### Impact of spot blotch, terminal heat stress and their combination on the parents *T. spelta* and *T. aestivum* and the population derived from them

Analysis of variance for the traits studied is presented in Table 1. ANOVA showed pronounced variation among the genotypes for all the studied traits. Furthermore, the interactions between the components of genotype × treatment (G × E) and genotype × treatment × year were observed to be significant. This indicates that genotypes had different responses for the treatments over different environments (years). Also, different treatments and their combinations had differential effects on various traits studied.

**Table 1:** Analysis of variance of 11 traits studied under different treatments and environments.

Source of Variation	d.f	AUDPC	BM	DH	GI	GPS	PH	PL	PY	SL	SPS	SS	TKW
Genotype	186	101,354.0*	2639.28*	259.25*	16.27*	222.33*	377.52*	139.21*	890.84*	6.83*	132.62*	419.61*	179.77*
Treatment	3	98,060,744.6*	299,620.45*	43,912.48*	14.27*	35,078.66*	35,518.55*	7129.93*	166,655.64*	147.08*	9155.14*	45,863.23*	27,641.68*
Year	2	1,072,244.4*	242,009.17*	801.22*	15.6	6346.42*	6037.95*	533.71*	23,499.18*	225.46*	529.02*	17,356.01*	5050.54*
Replication	1	5734.3	1737.94	2.5	19.11	161.85	374.99*	41.6	51.53	0.6	536.43*	0.17	0.01
Genotype × Treatment	558	27,151.5*	529.47*	8.75*	2.95*	38.92*	62.16*	24.64*	113.20*	2.13*	21.44*	186.97*	28.07*
Genotype × Year	372	16,488.1*	575.53*	8.01*	2.17	68.40*	85.92*	33.51*	138.82*	2.70*	17.88*	256.82*	39.65*
Treatment× Year	6	213,147.*	44,147.28*	2362.30*	34.25*	554.65*	20,892.51*	2489.83*	6123.84*	55.42*	341.99*	1676.28*	553.38*
Genotype × Treatment× Year	1116	8973.3*	390.72*	5.14*	2.08	29.93*	76.57*	25.12*	92.28*	2.18*	10.77*	185.44*	20.05*
Error	2242	3755.4	154.6	2.18	2.06	12.93	13.5	7.4	23.91	0.71	8.35	135.21	8.91

*Significant at *P* < 0.01. AUDPC, Area under disease progress curve; BM, 50 Tiller biomass; DH, Days to heading; GI, Glaucousness Index; GPS, Grains per spike; PH, Plant height; PL, Peduncle length; PY, Plot yield; SL, Spike length; SPS, Spikelets per spike; SS, Spikelet sterility; TKW, Thousand kernel weight.

Parent 1 (*T. spelta* H + 26) appeared to be a tolerant genotype as the magnitudes of trait deviations from control treatments were less for parent 1 compared to parent 2 (*T. aestivum* HUW234) under both the individual stresses and also under the combined stress (Table 2). The performances of the RIL population and parents for various physiological, agronomical, disease and yield traits under the three stress regimes (individually and their combination), are presented in Table 3a. The data clearly revealed that traits including DH, peduncle length (PL), plant height (PH), spike length (SL) and spikelets per spike (SPS) exhibited variation similar to the control under spot blotch stress; however, these traits showed major decline under both terminal heat and the combined stresses (Table 3b). The combined effect of both stresses reduced DH by 14.2%; PH by 11.5%; PL by 15.2% and SL by 4.3% compared to the control (Table 3b). Biomass (BM) was significantly reduced under all three regimes; plants exposed to the combined stresses showed the highest reduction (27.1%), while spot blotch stress or heat stress led individually to 13.2% and 20.6% reduction respectively. Average reductions in the number of grains per spike (GPS) were 13.0%, 19.1% and 25.6% under the impact of spot blotch, terminal heat stress and their combination. Spot blotch and terminal heat stress reduced TKW by 17.6% and 20.9%, respectively; however, the effect of combined treatment was far more detrimental with a decline of 31.8% compared to the control. The average PY decreased respectively by 21.9%, 27.7% and 39.0% under spot blotch, terminal heat stress and their combination. Similarly, SPS was reduced under all three stress treatments with maximum reduction shown by a combination of spot blotch with heat stress (11.4%), followed by heat stress (10.4%) and spot blotch (6.0%). All the three stresses—spot blotch, terminal heat stress and their combination—resulted in mean spikelet sterility (SS) increases by 6.8%, 9.2% and 14.9% respectively compared to control (Table 3b). Under control (fungicide protected) conditions, disease progress (AUDPC) was estimated to be very low, whereas under individual spot blotch stress, mean AUDPC was estimated to be 667.6 showing 256.4% increased disease progression over the control. Disease infection increased further to 781.9 under combined spot blotch plus terminal heat stresses, with 317.6% increase over the control. Overall, the combination of terminal heat and spot blotch had substantially higher impact on the tested wheat lines than the either single stress regime.

**Table 2:** Performance of parents *T. aestivum* (HUW234) and *T. spelta* (H + 26) under different stress treatments and environments.

Trait	Parent genotype	Treatments and environments			
		Control	Spot blotch	Terminal heat stress	Spot blotch and terminal heat stress
		(Timely sown protected with fungicide)	(Timely sown inoculated with pathogen)	(Late sown protected with fungicide)	(Late sown and inoculated with pathogen)
AUDPC	H + 26	117.87 ± 12.22	226.19 ± 7.61 **(91.89%)**	190.56 ± 7.48 **(61.67%)**	381.64 ± 6.97**(223.78%)**
	HUW 234	275.87 ± 12.26	847.12 ± **50.21 (207.07%)**	326.09 ± 27.34 **(18.20%)**	1106.78 ± 13.33 **(301.20%)**
BM (g)	H + 26	196.67 ± 14.14	190 ± 4.71 **(− 3.31%)**	176.67 ± 4.71 **(− 10.17%)**	168.33 ± 2.36 **(− 14.41%)**
	HUW 234	158.33 ± 2.36	141.67 ± 11.79 **(− 10.52%)**	116.67 ± 4.71 **(− 26.31%)**	116.67 ± 4.71**(− 26.31%)**
DH (d)	H + 26	111 ± 0	110.33 ± 1.41 **(− 0.60%)**	105.33 ± 1.41 **(− 5.12%)**	102.83 ± 0.24 **(− 7.36%)**
	HUW 234	71.50 ± 0.71	73 ± 1.41 **(2.10%)**	62.50 ± 0.24 **(− 12.59%)**	61 ± 0.47 **(− 14.68%)**
GI	H + 26	1.92 ± 0.35	1 ± 00 **(− 47.92%)**	1.50 ± 00 **(− 21.88%)**	1.50 ± 00 **(− 21.88%)**
	HUW 234	4.33 ± 0.24	2 ± 00 **(− 53.81%)**	4.25 ± 0.35 **(− 1.85%)**	2.75 ± 0.35 **(− 36.50%)**
GPS	H + 26	41.66 ± 0.32	41.43 ± 1.03 **(− 0.55%)**	37.58 ± 0.75 **(− 9.79%)**	36.43 ± 1.46 **(− 12.55%)**
	HUW 234	48.46 ± 1.42	41.08 ± 1.38 **(− 15.23%)**	39.60 ± 1.70 **(− 18.28%)**	31.97 ± 4.38 **(− 34.03%)**
PH ( cm)	H + 26	107.67 ± 1.41	108.50 ± 0.71 **(0.78%)**	104.17 ± 1.65 **(− 3.25%)**	100.17 ± 1.18 **(− 06.97%)**
	HUW 234	89.33 ± 3.77	87 ± 1.89 **(− 2.61%)**	79.17 ± 0.24 **(− 11.37%)**	75.67 ± 0.47 **(− 15.29%)**
PL (cm)	H + 26	34 ± 0.47	32.33 ± 0.94 **(− 4.91%)**	31.50 ± 0.24 **(− 7.38%)**	29.50 ± 0.24 **(− 13.24%)**
	HUW 234	36.17 ± 3.54	30.67 ± 5.66 **(− 15.21%)**	30.83 ± 0.24 **(− 14.76%)**	30.67 ± 1.89 **(− 15.21%)**
PY (g)	H + 26	52.71 ± 0.95	48.79 ± 0.69 **(− 7.44)%**	47.25 ± 0.25 **(− 10.36%)**	41.37 ± 0.11 **(− 21.51%)**
	HUW 234	86.72 ± 1.80	67.07 ± 2.59 **(− 22.66%)**	66.81 ± 0.58 **(− 22.96%)**	51.47 ± 0.25 **(-40.65%)**
SL (cm)	H + 26	12.50 ± 0.24	12.50 ± 0.24 **(0%)**	11.67 ± 00 **(− 6.64%)**	10.83 ± 0.71 **(-13.36%)**
	HUW 234	9.67 ± 00	10 ± 00 **(3.41%)**	8.67 ± 00 **(− 10.34%)**	9.33 ± 00 **(− 3.52%)**
SPS	H + 26	45.58 ± 1.58	44.28 ± .49 **(− 2.85%)**	41.89 ± 0.45 **(− 8.10%)**	39.77 ± 0.81 **(− 12.75%)**
	HUW 234	50.18 ± 0.59	48.72 ± 0.11 **(− 2.91%)**	47.52 ± 0.45 **(− 5.30%)**	45.81 ± 1.47 **(− 8.71%)**
SS (%)	H + 26	8.48 ± 3.87	6.37 ± 3.30 **(− 2.11%)**	10.08 ± 2.69 **(1.60%)**	8.07 ± 1.87 **(− 0.41%)**
	HUW 234	3.47 ± 1.74	15.40 ± 2.93 **(11.93%)**	16.79 ± 3.94 **(13.32%)**	30.13 ± 11.67 **(26.66%)**
TKW (g)	H + 26	24.37 ± 0.16	23.26 ± 0.13 **(− 4.56%)**	21.22 ± 0.26 **(− 12.93%)**	20.25 ± 0.66 **(− 16.91%)**
	HUW 234	44.78 ± 1.19	36.33 ± 3.13 **(− 18.87%)**	40.05 ± 1.25 **(− 10.56%)**	34.23 ± 0.93 **(-23.56%)**

The percent values in parenthesis represent the change from control.

**Table 3:** a Mean and range of various traits studied under different treatments and environments. b Effect of spot blotch, terminal heat stress and their combination on various traits studies in comparison to the control.

Trait	General statistics	Treatments and environments			
		Control (Timely sown protected with fungicide)	Spot blotch stress (Timely sown inoculated with pathogen)	Terminal Heat stress (Late sown protected with fungicide)	Spot blotch + Terminal heat stress (Late sown and inoculated with pathogen)
**a**					
AUDPC	Range	92.09— 304.30	226.19–899.74	158.65–347.69	381.64–1221.08
	Mean ± SD	194.53 ± 39.08	667.62 ± 109.67	245.10 ± 42.45	781.69 ± 122.02
BM (g)	Range	96.67—196.67	83.33–190.00	83.33–176.67	81.67–168.33
	Mean ± SD	140.31 ± 15.38	121.77 ± 14.27	111.37 ± 13.08	102.24 ± 9.66
DH (d)	Range	71.00–111	71.00—110.33	59.83–105.33	52.67–102.83
	Mean ± SD	77.08 ± 3.31	77.16 ± 3.42	66.34 ± 3.64	66.18 ± 3.55
GI	Range	1.00 –5.00	5.00–1.00	1.00–5.00	1.00–5.00
	Mean ± SD	2.48 ± 0.92	2.56 ± 1.00	2.44 ± 0.82	2.61 ± 0.80
GPS	Range	41.66–60.47	31.13–55.11	32.67–50.44	29.45–46.65
	Mean ± SD	51.16 ± 3.48	44.50 ± 4.15	41.37 ± 3.72	38.05 ± 3.67
PH (cm)	Range	69.33–107.67	74.00–108.50	63.17–104.17	64.50–100.17
	Mean ± SD	83.77 ± 4.98	84.01 ± 4.79	74.22 ± 4.75	74.10 ± 4.82
PL (cm)	Range	21.83–37.50	22.50–41.83	17.50–37.00	16.67–37.67
	Mean ± SD	29.33 ± 2.74	29.28 ± 2.83	25.00 ± 3.21	24.89 ± 3.11
PY (g)	Range	42.39–100.23	35.50–82.02	36.91–75.29	27.93–60.90
	Mean ± SD	74.40 ± 8.44	58.08 ± 7.72	53.77 ± 6.83	45.40 ± 5.28
SL (cm)	Range	8.00—12.50	7.83–12.50	6.50–11.67	6.83–11.50
	Mean ± SD	9.77 ± 0.73	10.04 ± 0.73	9.28 ± 0.78	9.35 ± 0.72
SPS	Range	45.58—64.50	43.68–60.24	41.89–57.77	39.77–57.51
	Mean ± SD	54.91 ± 3.12	51.60 ± 3.12	49.23 ± 2.56	48.63 ± 2.55
SS (%)	Range	1.40**–**15.77	1.19–37.87	3.79–30.55	5.35–38.48
	Mean ± SD	6.80 ± 3.08	13.64 ± 5.86	15.96 ± 5.58	21.74 ± 6.91
TKW (g)	Range	24.37–52.37	23.16–40.66	19.41–40.05	19.99–34.23
	Mean ± SD	37.67 ± 3.76	31.04 ± 3.08	29.78 ± 3.49	25.68 ± 2.83
**b**					
AUDPC	Mean	194.53	667.62	245.10	781.69
	% Change over control		256.35	29.49	317.61
	Range of change over control (%)		91.89 to 658.03	− 25.88 to 130.47	144.31 to 683.60
BM (g)	Mean	140.310	121.770	111.370	102.240
	% Change over control		− 13.21	− 20.63	− 27.13
	Range of change over control (%)		− 38.60 to 20.97	− 43.82 to 4.53	− 49.12 to − 6.90
DH (d)	Mean	77.080	77.160	66.340	66.180
	% Change over control		0.09	− 13.94	− 14.15
	Range of change over control (%)		− 9.36 to 10.39	− 19.14 to − 5.11	− 30.24 to − 3.46
GI	Mean	2.480	2.560	2.440	2.610
	% Change over control		3.51	1.56	5.56
	Range of change over control (%)		− 61.54 to 125	− 40 to 100	− 55.56 to 175
GPS	Mean	51.160	44.500	41.370	38.050
	% Change over control		− 13.01	− 19.13	− 25.63
	Range of change over control (%)		− 36.00 to − 0.54	− 34.76 to − 5.60	− 40.72 to –9.42
PH (cm)	Mean	83.770	84.010	74.220	74.100
	% Change over control		0.29	− 11.39	− 11.54
	Range of change over control (%)		− 14.86 to 21.88	− 22.47 to 0.72	− 26.86 to 7.21
PL (cm)	Mean	29.330	29.280	25.000	24.890
	% Change over control		− 0.16	− 14.75	− 15.15
	Range of change over control (%)		− 31.12 to 29.38	− 36.26 to 14.94	− 48.98 to 20.78
PY (g)	Mean	74.400	58.080	53.770	45.400
	% Change over control		− 21.93	− 27.72	− 38.97
	Range of change over control (%)		− 39.90 to − 5.47	− 45.57 to − 10.36	− 54.84 to 21.51
SL (cm)	Mean	9.770	10.040	9.280	9.350
	% Change over control		2.73	− 5.09	− 4.31
	Range of change over control (%)		− 24.32 to 33.96	− 24.19 to 14.81	− 32.79 to 23.21
SPS	Mean	54.910	51.600	49.230	48.630
	% Change over control		− 6.02	− 10.35	− 11.44
	Range of change over control (%)		− 23.52 to 11.29	− 22.29 to 2.66	− 26.90 to 4.05
SS (%)	Mean	6.800	13.640	15.960	21.740
	% Change over control		6.84	9.16	14.94
	Range of change over control (%)		− 3.37 to 30.54	− 4.12 to 24.30	− 0.41 to 31.67
TKW (g)	Mean	37.670	31.040	29.780	25.680
	% Change over control		− 17.60	− 20.94	− 31.82
	Range of change over control (%)		− 43.65 to − 4.53	− 42.46 to − 5.50	− 53.49 to − 11.43

### Correlation analysis under the combined treatment of spot blotch and terminal heat stress

**Figure 1:**
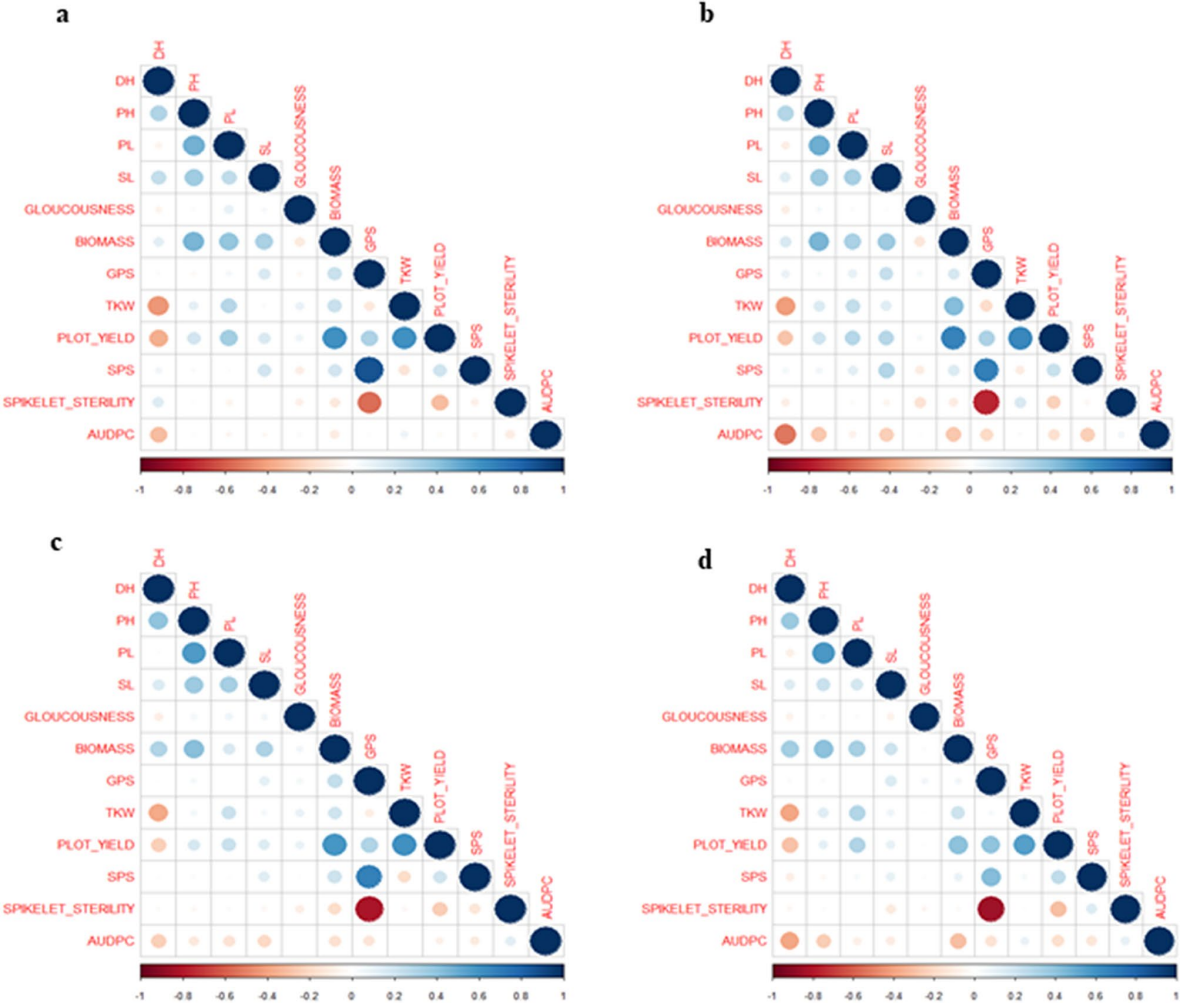
Correlation among traits in control (**a**); spot blotch (**b**); terminal heat stress (**c**); and combined stresses of spot blotch and terminal heat (**d**).

Correlation analysis among the studied traits was carried out separately for each stress regime including the control. The results of the correlation study under control treatment are presented in Fig. 1a while those under individual spot blotch, terminal heat stress treatment and combination of spot blotch and terminal heat stress are presented in Figs. 1b,c,d respectively.

In the following,** denotes *P* < 0.01 while * indicates *P* < 0.05. Under the combined stress condition, DH was positively correlated with PH (0.36^**^) and BM (0.34^**^). A highly significant negative correlation of DH was observed with AUDPC (− 0.39^**^), TKW (− 0.38^**^) and PY (− 0.28^**^). PH showed highly significant correlation with PL (0.58**), BM (0.4**) and SL (0.21**). Highly significant negative correlation (− 0.24**) was found for PH and AUDPC. PL was positively correlated with BM (0.32^**^), PY (0.29^**^), TKW (0.28^**^) and SL (0.17**). SL showed positive correlation with biomass (0.2^**^) and GPS (0.14^*^). BM showed a high degree of positive correlation with PY (0.41^**^) and TKW (0.21^**^). A significant negative association was found between BM and AUDPC (− 0.29^**^). GPS demonstrated a high positive correlation with SPS (0.42^**^) and PY (0.40^**^) while a very high degree of negative correlation was detected with SS (− 0.84^**^) and AUDPC (− 0.14^**^). TKW was found to be in a very high positive correlation with PY (0.54^**^). Significant negative association of PY was observed with SS (− 0.29**) and AUDPC (− 0.17*). SPS was positively correlated with SS (0.13^*^) while SPS showed a negative correlation with AUDPC (− 0.12); SS also showed a moderate positive correlation with AUDPC (0.09). The highest degree of negative correlation of AUDPC was found with DH (− 0.39^**^) followed by BM (− 0.29^**^), PH (− 0.24^**^), PY (− 0.17^*^) and GPS (− 0.14^*^). Glaucousness Index (GI) had little impact.

### Multivariate analysis

**Figure 2:**
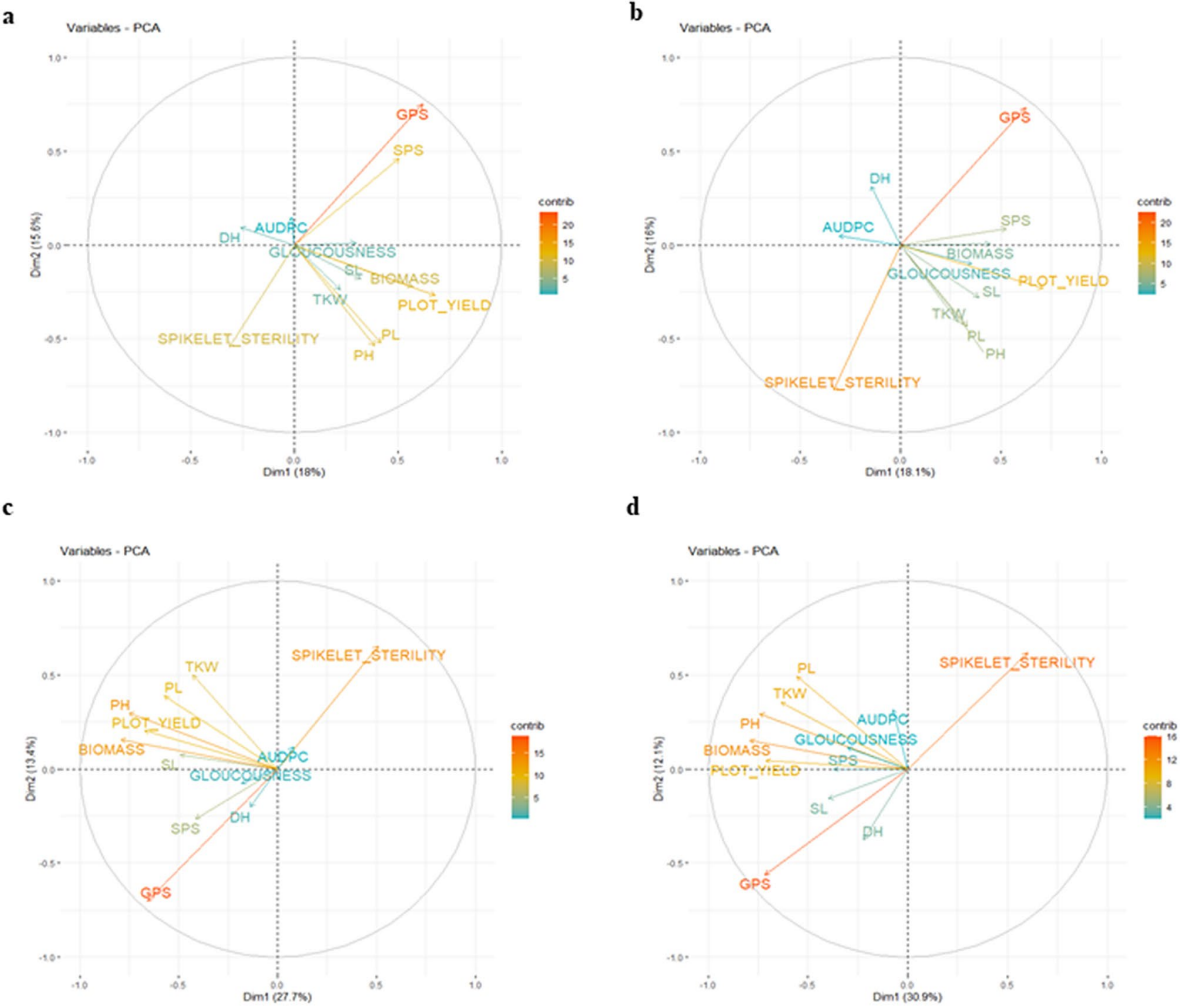
Principal Component Analysis Biplot for traits studied under control (**a**); spot blotch (**b**); terminal heat stress (**c**); and combined stresses of spot blotch and terminal heat (**d**).

Principal component analysis (PCA) showed eigenvalues > 1 for the first five components under the control environment. The first and second principal components (PC1 and PC2) explained a total of 33.6% phenotypic variation; PC1 and PC 2 explained 18.0% and 15.6% variations, respectively. Major contributors to these two PCAs were GPS, PY, SPS, BM, PL, PH and SS (Fig. 2a). Under spot blotch treatment, the first five principal components had eigenvalues > 1, where PC1 and PC2 explained 18.1% and 16.0% of the phenotypic variation. Thus, the first two principal components (PC1 and PC2) cumulatively explained 34.1% variation; major contributors were GPS, SS, PY, PH, PL, SPS, TKW, and SL (Fig. 2b).

Under terminal heat stress a cumulative phenotypic variation of 41.1% was explained by PC1 (27.7%) and PC2 (13.4%) where PCA demonstrated that the first five principal components had eigenvalues > 1. The major contributors to the first two PCs under terminal heat stress environment were GPS, SS, BM, PH, PY, PL and TKW (Fig. 2c). Under combined treatment of spot blotch and terminal heat stress, the first five principal components had eigenvalues > 1 with PC1 and PC2 explaining 30.9% and 12.1% phenotypic variation, respectively. The main contributors to the 43.0% variation explained cumulatively by PC1 and PC2 were GPS, SPS, BM, PH, PL, TKW and PY (Fig. 2d).

### Grain yield superiority and stress tolerance indices

Grain yield superiority and stress tolerance indices for the top five RILs compared to those for the best local variety HUW234 (parent 2), are presented in Table 4. All the top five lines for spot blotch tolerance showed Disease Tolerance Index (DTI) values significantly greater than that of HUW234, and had grain yields of 110.3–117.3% compared to the mean yield of HUW234. Similarly, all the top five lines selected based on Heat Tolerance Index (HTI) values showed yields of 103.7–111.6% compared to the mean yield of HUW234 under terminal heat stress. The data also showed that the top five lines that exhibited better tolerance to combined stress (terminal heat plus spot blotch), all had significantly superior Heat and Disease Tolerance Index (HDTI) value as compared to HUW234. Notably, four of these lines exhibited yields of 110.5–113.4% of the mean yield of HUW234 under the combined stresses. The top-ranked genotypes performing better under the individual stress of spot blotch disease as well as under the combined stress of spot blotch with terminal heat were lines 64, 71, and 139, while lines 64 and 175 had better tolerance to terminal heat stress alone as well as to the combined stress. Under simultaneous spot blotch and terminal heat stresses, lines 64, 71, 123, 139, and 175 were superior based on their tolerance indices. Of these, four lines—64, 71, 123, and 175—were also superior in performance to HUW234. Line 64 was the top performer against both of the individual stresses and the combined stress.

**Table 4:** Best RILs of *T. spelt*× *T. aestivum* based on grain yield under different stress treatments.

	Spot blotch						Terminal heat				Spot blotch and terminal heat					
Rank of genotypes	Genotype	DTI	AVG Yield (g)	Grain yield over local check (%)	AVG. AUDPC	AUDPC over local check (%)	Genotype	HTI	AVG Yield (g)	Grain yield over local check (%)	Genotype	HDTI	AVG Yield (g)	Grain yield over local check (%)	AVG AUDPC	AUDPC over local check(%)
1	64	1.48	78.7	17.3	568.69	− 32.87	89	1.31	78.2	11.6	64	1.10	60.9	10.5	640.29	− 42.15
2	21	1.26	77.1	14.8	485.08	− 42.74	64	1.23	76.1	8.6	123	0.90	60.9	10.6	725.48	− 34.45
3	139	1.21	74.2	10.6	558.16	− 34.11	58	1.22	72.7	3.7	175	0.88	62.5	13.4	527.46	− 52.34
4	32	1.20	74.1	10.4	488.22	− 42.37	175	1.19	75.3	7.4	71	0.88	62.3	13.1	509.31	− 53.98
5	71	1.18	74.0	10.3	421.76	− 50.21	32	1.13	76.0	8.4	139	0.88	53.8	− 2.3	634.06	− 42.71
P1	H + 26	0.47	58.7	− 12.4	226.19	− 73.30	H + 26	0.45	57.2	− 18.3	H + 26	0.40	50.3	− 8.6	381.64	− 65.52
P2	HUW234	1.05	67.1		847.12		HUW234	1.05	70.1		HUW234	0.81	55.1		1106.78	
C.D.(0.05)		0.10	4.6					0.10	4.9			0.10	4.0			
C.D.(0.01)		0.10	6.3					0.10	6.8			0.10	5.4			

The trait abbreviations are: DTI: Disease Tolerance index: HTI: Heat Tolerance Index: HDTI: Heat + Disease Tolerance Index.

## Discussion

In the present study, a RIL population derived from the cross *T. spelta* × *T. aestivum* was analyzed to identify possible superior wheat lines against spot blotch, terminal heat stress and combined spot blotch plus terminal heat stress. *T. spelta* was chosen as it has been identified as one of the most resistant genotypes for biotic and abiotic stresses^24^. It has also been demonstrated that *T. spelta* wheat lines possess higher activity of phenylalanine ammonia-lyase (PAL) and maintain a relatively high reactive oxygen species (ROS) content which allows them to survive better during fungal infections^25^. In addition, the ability of *T. spelta* lines to grow under limited oxygen supply makes them a potential source for improving the stress tolerance of wheat^22^. Our study was able to identify four *T. spelta*-derived wheat lines that performed well under both the experimental stresses and their combination, which further supports the use of *T. spelta* as a breeding parent for enhancing stress tolerance against spot blotch and terminal heat.

The present study has also provided detail insights about the impacts of spot blotch, terminal heat and their combination on various traits of wheat. All three stresses drastically reduced overall yield of the wheat lines tested. Interestingly, a few phenological and plant architecture-related traits like DH, PH, PL and SL were not greatly affected by the individual treatment of spot blotch. However, these traits were significantly reduced under the individual stress of terminal heat and the combined stress of spot blotch plus terminal heat. This emphasizes the fact that the detrimental effects of combined spot blotch and terminal heat stress are not necessarily additive on phenological and architecture-related traits; the reduction in these traits appears to be solely brought about by terminal heat stress. It has been proven that terminal heat stress is responsible for the abortion of anthers, decreased grain weight, reduced photosynthesis translocation and starch accumulation, thus decreasing overall wheat production^26,27^. The combination of spot blotch and terminal heat severely reduced PY followed by TKW, BM and GPS compared to individual treatments of heat or spot blotch. Spot blotch reduces the total photosynthetically active leaf area, which coupled with terminal heat stress stimulates premature leaf senescence too^28^. Heat stress causes disturbance in photosynthetic machinery and assimilate supply duration that ultimately lowers the yield^29^. Heat stress also causes oxidative stress damage through excess production of reactive oxygen species (ROS)^30,31^, which might be true even when heat stress occurs in combination with spot blotch. Occurrence of high temperature stress at meiosis results in impaired gametogenesis fertility, which causes reduced grain filling^30^. Thus, it is plausible that the suboptimal photosynthesis, poor photosynthates mobilization and reduced grain filling duration caused by the combination of spot blotch and terminal heat stresses is responsible for increasing sterility and reduction in grain yield and biomass accumulation. This finding is consistent with previous observations where a combined effect of spot blotch and terminal heat stress caused significant premature senescence of leaves, reduced grain filling, kernel weight and seed set^11,19,32,33^. However, future investigations are needed to elucidate the molecular and physiological aspects of combined effect of spot blotch and terminal heat stress.

The AUDPC for spot blotch was found to increase when the disease coincided with high temperature stress. Since spot blotch pathogen is more aggressive in late sown conditions, high temperature stress combined with spot blotch at the reproductive phase seems to be major factor that contribute to reducing wheat yield^32^. In agreement, our data shows that under the late sown condition an additive action of spot blotch and terminal heat significantly reduced wheat yield compared to the individual regimes of spot blotch or terminal heat. Since both these stresses have more impact when the wheat crop is in transition from vegetative to reproductive phase, they jointly hamper the grain formation and grain filling processes to reduce grain yield. Genotypes with early flowering capability can finish most of their reproductive processes before spot blotch and heat stress or their combination become intense. A negative correlation was reported between DH and spot blotch severity^34^, but the impact of terminal heat was not studied^35–37^.

In our study, all the stress treatments showed significant negative correlation of DH with PY and TKW. This supports the hypothesis that early flowering genotypes may perform better under spot blotch infection, heat stress and their combination, due to their potential to escape most of the stressed period^38,39^. SS is another trait that had a highly significant negative correlation with GPS and PY. Since the effect of both spot blotch and terminal heat become more severe at the reproductive stage, it is plausible that these stresses may be responsible for the production of structurally abnormal and non-functional florets^40^. A case of pollen abortion by heat stress has been reported in wheat^30^. Further, high-temperature stress at the anthesis and post-anthesis stages has been reported to cause a severe decline in GPS, through the hampering of spike growth and development, and by increased ovule abortion and pollen mortality^41–45^. Pollen cells and microspores are adversely affected by increased temperature stress at the reproductive stage in wheat, which leads to pollen sterility^46^.

We found some spelt-derived lines showing higher stress tolerance indices against spot blotch, terminal heat and their combination. RILs 64, 71, 123 and 175 were the genotypes most tolerant to spot blotch, terminal heat as well as to their combined stress. Interestingly, these four lines also gave higher yields under control conditions (no stress), suggesting that some genotypes have high stability and can respond well under various environment and stress conditions. This finding is in agreement with previous report where similar observations was reported for wheat lines under heat and drought stresses^47^. RIL64 was the top performer against all the three stress treatments and may be used in future breeding programs to enhance wheat stress tolerance in warm and humid regions of the world.

The present study has successfully utilized the potential of *T. spelta* in generating wheat lines with enhanced tolerance against spot blotch, terminal heat and their combination. The trait correlation study combined with multivariate analysis and tolerance indices provides an insight into potential selection criteria for wheat improvement against spot blotch, terminal heat and their combination. In the present study, four elite lines identified for their tolerance against these stresses may be used as parents in the future wheat breeding programmes.

## Methods

### Plant material and experimental conditions

A total of 187 genotypes—185 recombinant inbred lines (RILs) and their parents—were used in this study. The F_9-10_ RIL population was developed from the cross *T. spelta* (H + 26) × *T. aestivum* (cv. HUW234), where H + 26 was designated as P1 and HUW234 as P2. H + 26 is very resistant to spot blotch and is capable of withstanding high temperatures, while HUW234 is a variety widely adopted in the North Eastern Plains Zone (NEPZ) of India and shows significant spot blotch infection rates. HUW234 is a previously leading variety that covered around 4–5 million hectares in NEPZ of India during the 1990s; it was popular due to its high yield and excellent chapati (an Indian staple bread) quality. This variety continued to dominate the NEPZ until recently; it is believed still to account for about 1 Mha in NEPZ. The *T. spelta* parent was tested at Varanasi for two years and was found to show high resistance to spot blotch and yield higher under terminal heat stress.

The experimental material was evaluated at Agricultural Research Farm of Banaras Hindu University, Varanasi, India (25° 18′ N, 83° 03′ E and 75.5 m AMSL) for three consecutive years i.e. *Rabi* (winter) season 2013–14, 2014–15 and 2015–16. The mean temperatures (November–April) during these years were 25.4 °C, 23.2 °C and 24.5 °C respectively, while the annual rainfalls were 932.7, 1009.4 and 835.5 mm. The Varanasi Research Centre is known as a hot spot for spot blotch infection. All the 187 genotypes (RILs with parents) were sown in four different sets as follows:

(i) Timely sown protected control:
  Two replications of the population (185 RILs + parents) were sown in the third week of November and treated with a systemic fungicide, azoxystrobin, to protect them from the natural occurrence of spot blotch.
(ii) Timely sown disease inoculated/Spot blotch disease treatment:
  Two replications of the population (185 RILs + parents) were sown in the last week of November and inoculated with *B. sorokiniana*.
(iii) Late sown protected/Terminal heat stress treatment:
  Two replications of the population were sown in the last week of December and protected from the natural occurrence of spot blotch by spraying with a systemic fungicide, azoxystrobin.
(iv) Late sown disease inoculated/Combined spot blotch and terminal heat stress treatment:
  Two replications of the population were sown in the last week of December and inoculated with *B. sorokiniana*.

The experiment was laid out in an alpha lattice design with two replications. Each genotype was sown in two rows of two-meter-long plots under standard irrigated conditions maintaining a row-to-row distance of 20 cm and a plant-to-plant distance of 5 cm. The genotypes were allocated randomly within each replication using Fisher and Yates’ Random Table^48^. Recommended fertilizer doses (120 kg N: 60 kg P_2_O_5_: 40 kg K_2_O per ha) were applied in the field. The full amounts of K_2_O and P_2_O_5_ were supplied as a single dose at sowing, while nitrogen was provided in three split doses: 60 kg per ha at sowing, 30 kg at the time of the first irrigation [21 days after sowing (DAS)] and the remaining 30 kg at the time of second irrigation (45 DAS).

### Preparation and application of inoculum

The artificial epiphytotic condition was created by inoculation in the field with a virulent race of *B. sorokiniana*. An isolate of *B. sorokiniana* (HD 3069/MCC 1572) was obtained from the Department of Mycology and Plant Pathology, Institute of Agricultural Sciences, Banaras Hindu University, Varanasi and applied to create an artificial infection^49^.This isolate was purified and multiplied on potato dextrose agar (PDA) medium. Mass culture was produced on parboiled sorghum grains under aseptic conditions^50^. A spore suspension (10^4^ spore ml^−1^) was prepared and adjusted by soaking the colonized sorghum grains in distilled water, to which 100 μl Tween 20 per liter had been added. The inoculation was done at the ear emergence stage (GS 55)^51^ during the evening hours^52^. The field was irrigated immediately after inoculation to provide the proper humidity necessary for spore germination and disease development to take place.

### Data collection

Data were recorded for days to 50% heading (DH), PH, PL, SL, disease severity, GI, BM (50 randomly selected tillers), GPS (as the average number of grains from five randomly selected spikes), TKW, PY (as the yield from 50 randomly selected fertile tillers), SPS (as the average number of spikelets from five randomly selected spikes) and SS (as the average percentage of spikelets not setting seeds from five randomly selected spikes).

#### Disease scoring for calculating disease severity percentage and AUDPC

The quantification of spot blotch disease development was done by scoring at three different growth stages—GS 63 (beginning of anthesis to 50%), GS 69 (complete anthesis) and GS 77 (late milking)^53^. The scoring on each genotype was done on a double-digit scale (D1D2, 00–99)^52^. The scale’s first digit (D1) indicates vertical progress of disease on the plant, while the second digit (D2) indicates the severity of disease based on the total leaf area occupied by the disease symptoms. The disease score was later converted into disease severity (DS) in terms of percentage by using the formula as follows^54^:

$$\% {\text{ Disease}}\;{\text{Severity}} = \left( {{\text{D1}}/{9}} \right) \, \times \, \left( {{\text{D2}}/{9}} \right) \, \times {1}00$$

The corresponding disease severity percentages of each disease score taken at GS63, GS69 and GS77 were used for the area under the disease progress curve (AUDPC) calculation^55^ as follows:

$${\text{AUDPC }} = \mathop \sum \limits_{{{\text{i}} = 0}}^{{{\text{n}} - 1}} \left[ {\left\{ {\frac{{{\text{Yi }} + {\text{ Y}}\left( {{\text{i}} + 1} \right)}}{2}} \right\} \times \left( {{\text{t}}\left( {{\text{i}} + 1} \right){-}{\text{ ti }}} \right)} \right]$$

where Yi = disease level at time ti. {t (i + 1) – ti} = Time interval (in days) between two disease scores. n = number of dates at which spot blotch score was recorded.

#### Stress Tolerance Index (STI)

Stress tolerance index is an important parameter for understanding crop behavior under stress. The index was used to determine the tolerance of genotypes to stress and calculated by employing the following formula ^56^:

$${\text{STI}}\, = \,\left( {{\text{Ys }}*{\text{Yp}}} \right) \, /\left( {{\overline{\text{Y}}\text{p}}} \right)^{{2}}$$

where Yp=the average plot yield of particular genotype under non-stress conditions. Ys = the average plot yield of a genotype under stress conditions. Ῡp = the yield average of all genotypes under non-stress conditions

This was used to calculate the separate DTI (Disease Tolerance Index), HTI (Heat Tolerance index) and HDTI (Combined Heat and Disease tolerance Index). For DTI calculation, data from the timely sown disease (spot blotch) inoculated treatment was used. For HTI, data from the late sown protected/terminal heat stress (late sown protected) treatment was applied. Similarly, data from the late sown disease inoculated/combined spot blotch and terminal heat stresses (late sown disease inoculated) treatment was used for HDTI.

### Statistical analysis

The significance differences between treatments and the significance of differences among the genotypes were assessed by Analysis of Variance (ANOVA) using the proc GLM procedure of SAS statistical software version 9.4 (2014). In addition, ‘R’ software was used to perform the correlation analyses among the traits under study. The relationships between yield and other yield attributing traits were further explored using principal component analysis^57^. The results of the principal component analysis are presented using biplots constructed using the first two principal components for each treatment.
